# The Association between Mediated Deprivation and Ovarian Cancer Survival among African American Women

**DOI:** 10.3390/cancers15194848

**Published:** 2023-10-04

**Authors:** Andrew B. Lawson, Joanne Kim, Courtney Johnson, Kendra L. Ratnapradipa, Anthony J. Alberg, Maxwell Akonde, Theresa Hastert, Elisa V. Bandera, Paul Terry, Hannah Mandle, Michele L. Cote, Melissa Bondy, Jeffrey Marks, Lauren C. Peres, Joellen Schildkraut, Edward S. Peters

**Affiliations:** 1 Department of Public Health Sciences, College of Medicine, Medical University of South Carolina, Charleston, SC 29425, USA; 2 Usher Institute, School of Medicine, University of Edinburgh, Edinburgh EH16 4UX, UK; 3 Department of Biomedical Informatics, College of Medicine, Ohio State University, Columbus, OH 43210, USA; joanne.kim@osumc.edu; 4 Department of Epidemiology, Rollins School of Public Health, Emory University, Atlanta, GA 30322, USA; courtney.elizabeth.johnson@emory.edu (C.J.);; 5 Department of Epidemiology, College of Public Health, University of Nebraska Medical Center, Omaha, NE 68198, USA; 6 Department of Epidemiology and Biostatistics, Arnold School of Public Health, University of South Carolina, Columbia, SC 29208, USA; 7 Department of Oncology, Wayne State University School of Medicine, Population Studies and Disparities Research Program, Karmanos Cancer Institute, Detroit, MI 48201, USA; 8 Cancer Epidemiology and Health Outcomes, Rutgers Cancer Institute of New Jersey, New Brunswick, NJ 08625, USA; 9 Department of Medicine, University of Tennessee Medical Center-Knoxville, Knoxville, TN 37920, USA; 10 Bren Simon Comprehensive Cancer Center, Indiana University Melvin, Inidianapolis, IN 46202, USA; mlcote@iu.edu; 11 Department of Epidemiology and Population Health, College of Medicine, Stanford University, Stanford, CA 94305, USA; 12 Department of Surgery, Duke University School of Medicine, Durham, NC 27710, USA; jeffrey.marks@duke.edu; 13 Department of Cancer Epidemiology, H. Lee Moffitt Cancer Center and Research Institute, Tampa, FL 33612, USA; lauren.peres@moffitt.org

**Keywords:** ovarian cancer, survival, deprivation indices, Kolak, Singh, Yost, CDI, causal mediation, Bayesian, structural equation, mixed effect models

## Abstract

**Simple Summary:**

Deprivation indices (DIs), constructed from area-level socio-economic indicators, allow to assess the acknowledged relationship between socio-economic status and health outcomes. Their role in adjusting for deprivation has not been evaluated within a mediation analysis of ovarian cancer survival. In this paper, we present a Bayesian SEM approach to causal mediation for direct and indirect effects of DIs in the context of a cohort of African American women with ovarian cancer.

**Abstract:**

Background: Deprivation indices are often used to adjust for socio-economic disparities in health studies. Their role has been partially evaluated for certain population-level cancer outcomes, but examination of their role in ovarian cancer is limited. In this study, we evaluated a range of well-recognized deprivation indices in relation to cancer survival in a cohort of self-identified Black women diagnosed with ovarian cancer. This study aimed to determine if clinical or diagnostic characteristics lie on a mediating pathway between socioeconomic status (SES) and deprivation and ovarian cancer survival in a minority population that experiences worse survival from ovarian cancer. Methods: We used mediation analysis to look at the direct and indirect causal effects of deprivation indices with main mediators of the SEER stage at diagnosis and residual disease. The analysis employed Bayesian structural equation models with variable selection. We applied a joint Bayesian structural model for the mediator, including a Weibull mixed model for the vital outcome with deprivation as exposure. We selected modifiers via a Monte Carlo model selection procedure. Results: The results suggest that high SES-related indices, such as Yost, Kolak urbanicity (URB), mobility (MOB) and SES dimensions, and concentrated disadvantage index (CDI), all have a significant impact on improved survival. In contrast, area deprivation index (ADI)/Singh, and area level poverty (POV) did not have a major impact. In some cases, the indirect effects have very wide credible intervals, so the total effect is not well estimated despite the estimation of the direct effect. Conclusions: First, it is clear that commonly used indices such as Yost, or CDI both significantly impact the survival experience of Black women diagnosed with epithelial ovarian cancer. In addition, the Kolak dimension indices (URB, MOB, mixed immigrant: MICA and SES) also demonstrate a significant association, depending on the mediator. Mediation effects differ according to the mediator chosen.

## 1. Introduction

In recent years, deprivation indices (DIs) have been widely used in population-based epidemiologic studies to examine the upstream area-level impacts of societal contexts on health risk. These indices are usually constructed from socio-economic indicators, available at specific area-level measures, and are often used to assess the relationship between area-level socio-economic status (SES) and health outcomes. DIs differ based on the number of variables included, variable selection method, and conceptual constructs represented. A recent extensive survey of deprivation indices in cancer studies found that 24 commonly used indices were associated with a range of cancer types and outcomes, including incidence, survival, and mortality [1]. In that review, a small number of indices, such as Yost, Area Deprivation Index (ADI), and Concentrated Disadvantage Index (CDI) were most frequently used.

In this study, we have sought to assess the importance of a range of DIs in relation to the survival experience of African American women diagnosed with ovarian cancer. We examined a cohort of ovarian cancer patients from the African American Cancer Epidemiology Study (AACES) [2]. For this purpose, we have selected a range of DIs which are typically employed and also a few recently developed indices which have yet to be extensively evaluated. Our goal is to evaluate the role of DIs in relation to ovarian cancer survival while adjusting for known prognostic factors such as stage at diagnosis and residual disease. Our analytic approach focuses on innovative mediation methods to accommodate modifiers and extra variation via a Bayesian hierarchical joint model framework. In a companion paper, we have examined different mediators such as histology and diagnostic delay, and examined a more restricted set of DIs but also included an evaluation of segregation indices [3].

## 2. Methods

### 2.1. Study Population

The AACES study (Phase 1) included 11 recruitment sites to provide geographic diversity, selected based on regions with large African American populations: Alabama, Georgia, Illinois, Louisiana, metropolitan Detroit (Michigan), New Jersey, North Carolina, Ohio, South Carolina, Tennessee, and Texas [2]. Rapid-case-ascertainment (RCA) was used to identify epithelial ovarian cancer (EOC) cases through state cancer or Surveillance, Epidemiology, and End Results (SEER) registries, and through gynecologic oncology departments at individual hospitals. After identification by the registry, the central study team contacted participants via phone to administer the baseline survey questionnaire. Further detail on enrollment has been described elsewhere [2]. Eligible cases had histologically confirmed EOC and were centrally reviewed by an expert study pathologist. Additional eligibility criteria included women who self-identified as Black or African American, age 20–79 at the time of diagnosis, and able to complete an interview in English. Participants were recruited between December 2010 and December 2015. As of 2021, the average and median survival time for all phase 1 cases was 4.79 years and 4.81 years, respectively. The survival rates for this cohort are consistent with the observed survival rates using national SEER data [4], when the condition is surviving at least 10 months past diagnosis to account for the fact that rapidly fatal cases do not make it into the study, even when employing RCA. During AACES Phase 1, we attempted to contact 1720 cases, 70% of which (*n* = 1199) were actively reached. Of these, 592 (49%) were successfully interviewed and 388 (32%) actively refused. The remaining 219 patients were not enrolled for reasons such as ineligibility and passive refusal. Approximately 50% of the phase 1-enrolled cases participated in at least one follow-up survey. Phase 2 is actively recruiting additional EOC cases, but these are excluded from the current analysis due to the brief follow-up time.

### 2.2. Deprivation

Deprivation indices are derived measures typically based on census socio-economic indicators and are used to estimate the areal socio-economic context of an individual or neighborhood. They are usually available within a range of small area units such as census block groups (BGs), census tracts (CTs) or postal/ZIP Codes. Previous studies of breast cancer, lung cancer, and prostate cancer have reported consistent relationships between neighborhood disadvantage or deprivation and worse survival [5,6,7,8].

The census tract was chosen as the spatial resolution level for indices examined here (see Section 2.3 for discussion). The following is a description of the chosen census tract DIs and their rationale for use.

#### 2.2.1. Yost Index

The Yost index is an SES index constructed via principal component analysis of seven variables [9,10]. The variables included are education level, 2 income variables, 2 housing variables, unemployment rate and blue collar proportion. It is widely used in population-level cancer studies and is available at various aggregation levels via the SEER database program (https://seer.cancer.gov/seerstat/databases/census-tract/index.html (accessed on 21 October 2019)). A higher Yost value represents a higher SES level. We have used the index computed for a range of years, 2006–2010, which represents the contextual census information closest to the year range for the study population.

#### 2.2.2. Singh Index (ADI)

The Singh ADI is a multidimensional composite index using 17 measures of SES selected through factor analysis and principal component analysis [11,12]. The components of the ADI include census variables related to education level, employment/occupation, income/poverty, housing, socio-demographics, and mobility. The components with the highest relative weights were those related to poverty, income, and education. The index was standardized to have a mean of 100 and a standard deviation of 20. A higher value of ADI represents more deprivation and can be inspected in a continuous or categorical manner. This index was computed from the 2010 census.

#### 2.2.3. Kolak Measures

These measures were recently introduced and applied at the CT level based on a framework of social determinants of health (SDOH) [13]. In this approach, fifteen variables characterizing SDOH, based on a 5-year mean from the 2014 American Community Survey, were analyzed and simplified to four indices using factor analysis and principal component analysis in a manner similar to the construction of the ADI. Each index represents the following components: socioeconomic advantage (SES: low poverty, higher education status, and insurance coverage), limited mobility (MOB: age, persons with disabilities, and presence of children), urban core opportunity (URB: income, renting population, rent burden, and lack of vehicles), and mixed immigrant cohesion and accessibility (MICA: immigrant or multilingual and crowded housing).

#### 2.2.4. Concentrated Disadvantage Index (CDI)

This measure of concentrated disadvantage includes variables related to public assistance, unemployment, poverty, female-headed households, African American population, and children. These variables were selected through factor analysis, summed, and then standardized to create the final index, with a higher CDI representing more disadvantage. Hossain et al. [5] explored the role of CDI and triple-negative breast cancer. They found associations with the racial disparities of both later stage at diagnosis and poorer survival. CDI was computed from the 2010 census and 2006–2010 ACS.

#### 2.2.5. Percent under the Poverty Line (POV)

Krieger et al. [14] performed a thorough comparison of several variables measuring SES and found that measures capturing deprivation (such as the Townsend index) were the most effective. Specifically, they used the commonly applied single variable of the percentage of persons below poverty (census defined) as well as more complex composite indices. POV was measured using the 2010 census.

### 2.3. The Spatial Context

The spatial context of deprivation is important as the influence of indices could operate at various spatial scales. In this study, we limit our focus to CTs only to allow for consistency across the available measures. The Kolak SDOH indices are not available at the BG [13], and the ZIP Code is based on postal delivery, and therefore inconsistent in terms of the size of the population covered.

The baseline residential addresses of Phase 1 participants were geo-coded to the CT level. The AACES Phase 1 sample is sparse across a selection of US states, and thus the spatial context is limited. The contextual environment has to be leveraged to provide any neighborhood reference. Hence, in the analyses, we exploit a rich class of Bayesian hierarchical models (BHMs) which include predictors and random effects to allow for contextual adjustment. This approach includes observed contextual predictors and also unobserved contextual effects that account for extra variation.

### 2.4. Bayesian Mediation Methodology

To assess the impact of DIs on the ovarian cancer survival experience of our study participants, we adopted a Bayesian causal mediation approach [15,16,17], creating separate models for each index or SDOH measure. This approach assumes a structural equation model (SEM) whereby any mediator is modeled jointly with the outcome. In these joint models, the outcome is assumed to be a function of exposure, mediator, modifiers, and a random effect representing unobserved confounding or extra variation. One of the major advantages of BHMs is the ability to incorporate unobserved variation via random effects [18]. The mediation model structure [19,20] allows for the estimation of direct and indirect effects of predictors; hence, it is possible to examine the paths within the directed dependence graph.

A summary of the approach is given here, with details of the approach and estimation method available in Supplementary Materials File S1. For a given survival outcome *T*, we specify a conditional model T|X,M,C,U, where *X* is the exposure, *M* is the mediator, **C** is the set of modifier variables, and *U* is the random term representing unobserved confounding.

We assume a conventional Weibull model for the survival outcome, conditional on the mediator, exposure, modifiers and unobserved confounding. This choice of model allows for a flexible form of hazard. The log of the hazard is furnished with a linear predictor representing the modifier set.

The mediation model is defined as conditional on exposure and modifiers. Note that the same random effect is shared and scaled in each outcome and mediator model. Finally, a random effect model is assumed, which, in general, can depend on the exposure and modifiers.

In the above model, the linear predictor for modifiers is usually assumed to be fixed. However, it is useful to consider an extension whereby we examine a range of putative modifier combinations during the estimation process. Gibbs variable selection is a Bayesian method that allows for the sampling of all models within the computational method of Markov Chain Monte Carlo (MCMC) [21,22,23]. MCMC is an iterative simulation-based method that ultimately provides samples from the posterior distribution of the joint model. This joint posterior distribution is summarized to provide estimates of important quantities, such as parameter means and variances. With Gibbs variable selection, different modifiers are randomly selected for inclusion in or deletion from the model within the MCMC iterations. How often a modifier is included in the model is recorded. This provides evidence for the importance of the modifiers.

## 3. Mediation Models as Applied in AACES

**Figure 1:**
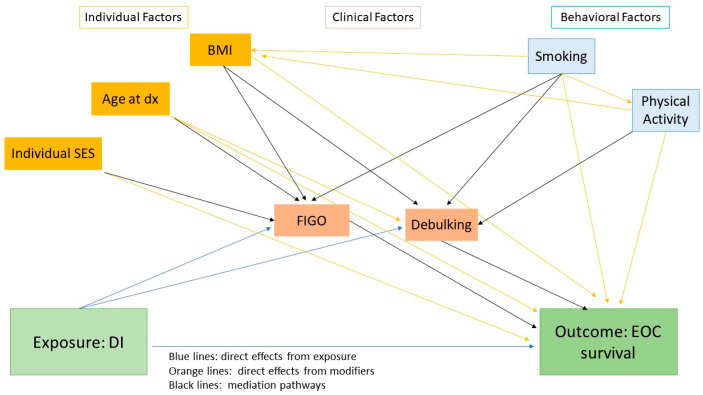
Potential relations between ovarian cancer survival and associated mediators and modifiers. Note that Stage and Debulking Status have not been depicted with a link as they are analyzed separately in this work.

In Figure 1, we depict the hypothetical relations between the survival outcome and sets of potential mediators and modifiers. Those chosen in this analysis are discussed in detail in the following sections.

### 3.1. Outcome

We consider the vital outcome, survival of the cohort participants, as our focus. Here, survival is defined as the time in days from diagnosis to the date of death or last contact, updated in 2021 based on the National Death Index, LexisNexis, and registry updates. This is consistent with our previous study [3] and allows us to compare it with findings from that study.

### 3.2. Exposures

The focus of this study is on the role of deprivation indices in EOC survival. As DIs are usually included as single parameters in models, we assumed that each index should be treated as the exposure and fitted separately with the mediator and modifier sets for our evaluation. As described above, these exposures were derived using census, ACS data, or SEER databases.

### 3.3. Mediators

Potential mediators were chosen a priori based on existing knowledge of their importance in EOC survival, and their values were abstracted from pathology reports. In this analysis, stage is a combination of FIGO (International Federation of Gynecology and Obstetrics) staging and SEER summary stage, considering localized, regional, or distant values. As there are 3 levels of stage, the indirect effect of the stage mediator is computed for each level separately.

Residual disease after surgery is the strongest predictor of survival among women with ovarian cancer [24,25]. However, residual disease is not consistently reported in medical records and results in large amounts of missing data. Debulking status is correlated with residual disease and may also be found in medical records. Therefore, we used debulking status for this analysis. We created an algorithm that mapped residual disease and post-treatment levels of CA125, indicative of residual disease, into a variable for optimal versus suboptimal debulking status to reduce the effect of missing data. Here, post-adjuvant chemotherapy levels of CA125 < 35 was classified as optimal debulking, and post-adjuvant levels of CA125 ≥ 35 was classified as suboptimal debulking. The White–Royston method of multiple imputation with study site, histology, stage, receipt of adjuvant therapy, receipt of neoadjuvant therapy, and age at diagnosis was used to impute the remaining missing 190 datapoints over 25 datasets [26]. Comparison of the hazard ratios (HRs) and credible intervals (CIs) from the complete case analysis to the imputed datasets shows similar results, thereby justifying this imputation. This was further justified with the fact that the survival curve of those missing debulking status fell between those with optimal debulking and those with suboptimal debulking; this suggested that debulking status was truly missing at random (MAR). To obtain an individual observation of debulking status, the mode value of debulking status for each participant was selected as the mode across imputations within participant.

### 3.4. Modifiers

We examine the same range of modifiers that we believe to be relevant in the analysis of EOC survival outcomes as included in our companion paper [3]: BMI, smoking status, physical activity, age at diagnosis, and self-reported gross annual family income. All data were ascertained from a baseline interview questionnaire that the participants complemented. BMI was calculated as kg/m^2^ from self-reported height and weight at a year prior to diagnosis. Smoking status was categorized as never, former, or current smoker. Physical activity was defined as the amount of weekly physical activity reported in the year prior to diagnosis and was further categorized to capture inactivity. The 2008 Physical Activity Guidelines for Americans (PAGA) was employed whereby activity is specified as 75 min of weekly strenuous activity, 150 min of weekly moderate activity, or an equivalent combination of both. The categories are yes vs. no. Self-reported family income was collected in categories of less than USD 10,000, USD 10,000–USD 24,999, USD 25,000–USD 49,999, USD 50,000–USD 74,999, USD 75,000–USD 100,000, and more than USD 100,000.

## 4. Results

Basic descriptive summaries of the selected clinical characteristics of the cohort are presented in Table 1, and summaries of the DIs and modifiers are shown Table 2.

To frame the discussion of the DIs and their role, we note the following about their direction of effect. First, large values of POV, CDI, MICA and ADI are indicative of increased deprivation, whereas large values of Yost, URB, MOB and SES are associated with high SES in a given spatial unit. Hence, the direction of survival effects will differ depending upon the index employed.

For stage as a mediator, in Table 3 we observe that age is associated with URB, MICA and ADI, whereas BMI is only marginally associated with any DI. Self-reported income is associated with the MOB index but marginal with others. Smoking seems relevant to MICA, the mixed immigrant index, while physical activity is relevant to all the indices. For URB, the direct estimated effect of the DI is 0.731, and the 95% credible interval (CI) is bounded below 1 (0.560, 0.914). This is also true for the MOB and SES indices. However, the MICA and ADI indices are not well estimated (the 95% CI crosses 1). The indirect effects are not well estimated and because of this the total effect is not well estimated.

In Table 4, age and physical activity are relevant for Yost 2010 and POV, and smoking is relevant to CDI. In the parameter estimates, it appears that only the direct effect of Yost 2010 (0.736; 95% CI: 0.566, 0.954) is well estimated and again the indirect effects have very wide CI. With stage as a mediator, only the Kolak URB, MOB, SES measures and YOST yield reduced hazard and increased survival.

Table 5 and Table 6 describe the mediation effect of debulking status. For the selected modifiers, age is selected for most indices, except MOB and MICA, whereas BMI is selected for ADI, YOST and POV. Smoking is selected for MICA, whereas physical activity is selected only for MOB and SES. The indices found to be well estimated were URB, MOB, SES, MICA, ADI and CDI. YOST only narrowly crossed 1.0 (0.816; 95% CI: 0.664,1.002). Due to the wider CI for the indirect effects, none of the total effects are well estimated here.

Throughout, the consistently well-estimated indices are URB and SES from the Kolak set, whereas Yost 2010 is also well estimated, with the exception of the debulking status mediator where it narrowly crosses 1.0 (0.664, 1.002). POV has a poorly estimated direct effect (1.751; 95% CI: 0.763, 4.508) and wide limits on indirect effects. In some cases, indirect effects have very wide credible intervals and so the total effect is not well estimated despite estimation of the direct effect.

## 5. Discussion

Our findings suggest a number of important considerations for research on the impact of neighborhood deprivation measures in relation to ovarian cancer survival. We observed evidence that DIs were associated with survival, although the magnitude and precision of the associations varied considerably. The most marked direct effect is observed with the Kolak URB measure when debulking status is the mediator. In contrast, when stage is the mediator, the three Kolak measures, URB, SES and MOB, and Yost, all show marked reduction in hazard, and hence increased survival. 

Of the high SES measures, the Yost and the Kolak URB dimension show the strongest magnitude of associations with higher area-level SES associated with increased survival. Ross et al. [9] used the Yost index to explore racial disparities in ovarian cancer survival and observed that while SES may be associated with survival disparities, these disparities still remained when adjusting for the Yost index. Hodeib et al. [27] reported that the Yost index was also a predictor of treatment adherence for women with early stage ovarian cancer. Notably, the direct poverty measure (POV) was not significantly associated with ovarian cancer survival. 

There are few additional studies of ovarian cancer using deprivation indices. An exception is Hufnagel and colleagues [28] where an increase in ADI was found to negatively affect ovarian cancer survival. In our study, ADI was found to be associated with shorter survival; the relation was not significant with mediation by stage but was found to significantly increase hazard when mediated by debulking status. This may suggest that a relation exists between ADI and stage. A significant association has been reported between ovarian cancer-specific survival and a concentrated disadvantage factor (CDI) [29,30], which extends the disparity in survival between Black and White women. Higher concentrated disadvantage was associated with poorer survival. In our study, we similarly observed that elevated CDI was significantly associated with poorer survival, albeit when debulking status as the mediator. In a previous analysis of ovarian cancers’ clinical markers (debulking status, late stage at diagnosis and high grade serous), CDI was found to be significantly associated with these outcomes at the CT level [29]. In subsequent work [31], an association was also found between a census tract CDI and racial survival disparity in ovarian cancer within a single large county in Illinois.

In general, in this study, high SES was observed to be significantly associated with longer survival in most of the SES indices (Yost 2010, URB, MOB, and SES), whereas for the deprivation indices (POV, CDI, MICA, and ADI), higher deprivation was observed to be associated with shorter survival. In this latter group, MICA, ADI and CDI were well estimated under debulking status as mediator. However, ADI was not well estimated under stage as a mediator. It is also notable that POV was not found to be well estimated under any mediator in our study. For CDI, the biggest change was under debulking status, whereas with stage as mediator, it is not well estimated.

However, it should be noted that the mediation results cited are conditional on the presence of selected modifiers. For example, with stage as the mediator, the inclusion of age and physical activity led to a significant URB hazard reduction, as in the case of YOST, whereas BMI played a significant role in ADI, POV and Yost under either stage or debulking status as mediators. The importance of BMI and physical activity in relation to the role of inflammation has been noted for this cohort [32]. It is also notable that self-reported income does not appear to be strongly associated with most of the indices examined as it was only marginally associated with the MOB index under stage mediation. However, MOB is significantly associated with reduced hazard when self-reported SES is included. The details of future analyses are likely to vary between study populations, but the general point is that the details of model selection can have important impacts on the results. Finally, there are a number of caveats to these general conclusions. First, all analyses were based on CT level-derived measures, and so results could vary if BG or county were the spatial unit chosen. Our cohort is limited to self-identified Black women with ovarian cancer and so racial disparity is not our focus, although there are results reported previously that ethnic disparity in survival for ovarian cancer was significantly associated with neighborhood disadvantage [30]. There may also be some inherent selection bias in our study sample. Women with the rapidly fatal ovarian cancer diagnoses may not be represented, as many of the women identified through RCA were deceased upon attempted contact. Additionally, the women who declined participation may have done so for various reasons, including feeling too ill or being warier of medical research. Based on this, the relationship between area-level deprivation and the most severe diagnoses of ovarian cancer are yet to be fully understood.

Our mediation analysis did not examine multiple mediation [33] as we confined our focus to single mediators. While multiple mediation is certainly feasible within our strategy, our focus on evaluating DIs limited our approach. In future work, we intend to examine multiple mediation and effect modification. Our results are conditional on the inclusion of selected important modifiers (mainly age and BMI) but also included random effects which allowed for unobserved confounding, and any analysis which did not adjust for these predictors would likely have extra noise. Inclusion of random effects within the paradigm is therefore important in allowing for this extra variation. Some alternate modifiers which were not included were education level (found to be confounded with SES), insurance coverage, comorbidities, and dietary assessment. Additional considerations pertain to information bias and selection biases. As some measures are self-reported, we may have misclassification bias. However, the direction of this bias is not clear. As many of the modifiers are categorical, it is possible that random biases are averaged. As we examined a subset of the cohort, it is possible that some results reflect that selection. These are the acknowledged limitations of this study.

## 6. Conclusions

This mediation-based analysis of DIs has wide-ranging implications for both neighborhood effects on ovarian cancer survival, and on the relative effectiveness of these indices when used in conjunction with different mediators and sets of modifiers. First, it is clear that the commonly used indices such as Yost, ADI, or CDI all have a significant impact on the survival of Black women diagnosed with EOC. In addition, the relatively new Kolak indices (URB, MOB, MICA and SES) also demonstrate a significant association under different mediators, particularly with URB and SES, which are consistently associated with a reduction in hazard across mediators.

Mediation effects differ by the mediator chosen. In some associations, the indirect effects have a wide CI, where the total effect is not well estimated despite an estimation of the direct effect. This suggests that mediation in this case may not add additional information to describe the relationship between these indices and survival. The stage and debulking status mediators also display large indirect CIs. This contrasts with the previously reported results [3], where histology and diagnostic delay, which displayed shorter indirect intervals, had well-estimated total effects. It appears that with histology and diagnostic delay, mediation is potentially more important in the relationship between these indices and survival than stage or debulking status. 

If the research focuses on identifying and adjusting for potential factors that confound relations in ovarian cancer survival studies, then the deprivation indices should be considered as important general adjusters. In terms of modifiers, age and BMI, or age and physical activity were selected often within our strategy, whereas self-reported SES or smoking do not often appear as important modifiers when included with other modifiers. Also, different mediators can affect the degree of adjustment achieved by use of DIs, and SES-based indices, such as Yost, ADI, URB, MOB, or SES, are much more effective in explaining survival variation than others examined here. While stage, our primary mediator, is important in explaining survival variation [34], it behaves differently when mediating the effects of different DIs. The indices with the largest impact on survival appear to be URB, SES, and Yost among the high SES indices, whereas ADI yields higher impacts than CDI among the high deprivation indices. The null results for POV indicate that a simple poverty measure (such as % population below the poverty line) does not provide significant adjustment in survival.

In summary, the hypothesis that area-level deprivation would be associated with worse prognosis was supported by the observations that several commonly used deprivation indices, such as Yost, ADI, or CDI, were significantly associated with epithelial ovarian cancer survival in a cohort of Black women. We also observed that the impact of mediators varied according to the deprivation index and the mediators considered.

## Figures and Tables

**Table 1:** Selected clinical characteristics of the cohort (*n* = 558). The table count only reflects the inclusion of participants whose geocoding was complete.

Characteristics	Mean (SD)	
Days on Study	1774.50 (962.86)	
	N	Percentage (%)
Survival Status		
Alive (censored)	221	39.6
Deceased	337	60.4
Stage		
1-Localized	125	23.9
2-Regional	51	9.8
3-Distant	346	66.3
Unknown	36	-
Debulking status—after imputation		
1 = Optimal debulking (or CA125 after adjuvant <35)	391	70.1
2 = Suboptimal debulking (or CA125 after adjuvant ≥35)	167	29.9

**Table 2:** Selected characteristics of the DIs and modifier variables. (*n* = 558).

Characteristics	Mean (SD)	
Age	58.04 (10.90)	
BMI	32.82 (8.42)	
Kolak measures		
URB	−0.40 (0.85)	
SES	−1.20 (2.10)	
MOB	−0.48 (1.41)	
MICA	0.48 (1.00)	
Yost 2010	9493.66 (921.62)	
ADI	108.84 (19.99)	
Percentage of people live below federal poverty level (POV)	0.17 (0.13)	
Concentrated disadvantage index (CDI)	0.00 (3.92)	
Characteristics	N	Percentage (%)
Self-reported income		
less than $10,000	113	22.2
$10,000–$24,999	120	23.6
$25,000–$49,999	125	24.6
$50,000–$74,999	76	14.9
$75,000–$100,000	44	8.6
More than $100,000	31	6.1
NA	49	-
Smoking status		
Never smoker	309	55.4
Ever Smoker (Former/current)	249	44.6
PAGA **		
1 = Yes	130	25.1
2 = NO	387	74.9
NA	41	-

Number of missing observations for the continuous variables. Kolak measures: 1 each, Yost: 13, ADI: 8, POV: 8, BMI: 3. ** Physical activity guidelines for Americans (2008) yes: active, no: inactive.

**Table 3:** Bayesian analysis of DI on ovarian cancer survival mediated by cancer stage (3 levels). Each column represents a separate model with a different DI as exposure. Well-estimated hazard ratio shown in bold.

Gibbs Variable Selection for Covariates (Modifiers). Covariates with Values Higher Than 0.5 Will Be Included in the Analysis					
Mediator: Stage	URB	MOB	SES	MICA	ADI
Age	**1**	0.458	0.002	**0.912**	**1**
BMI	0.457	0.475	0.144	0.499	0.495
Self-reported SES	0.295	**0.509**	0.058	0.488	0.483
Smoking	0.428	0.491	0.033	**0.506**	0.489
Physical activity	**0.530**	**0.516**	**1**	**0.504**	**0.511**
Analysis result with selected modifiers. Each cell shows a posterior mean and its 95% credible interval					
Direct effect (Deprivation indices)	**0.731 (0.560, 0.914)**	**0.887 (0.78, 0.999)**	**0.895 (0.808, 0.991)**	1.149 (0.916, 1.437)	1.873 (0.665, 5.456)
Indirect effect Through stage 1	0.674 (0, 4.5 × 10^12^)	0.983 (0.221, 3.977)	0.996 (0.188, 4.571)	1.022 (0.258, 4.088)	0.979 (0.141, 6.51)
Indirect effect Through stage 2	0.683 (0, 8.5 × 10^11^)	0.995 (0.493, 1.874)	1.012 (0.508, 2.013)	0.987 (0.381, 2.476)	0.980 (0.352, 2.595)
Indirect effect Through stage 3	0.711 (0, 1.06 × 10^11^)	0.998 (0.120, 6.812)	1.028 (0.086, 18.340)	0.902 (0.037, 12.862)	1.024 (0.125, 8.055)
Total effect 1 (Direct + Indirect through stage 1)	0.492 (0, 3.1 × 10^12^)	0.872 (0.198, 3.572)	0.891 (0.169, 4.112)	1.175 (0.292, 4.808)	1.833 (0.214, 16.031)
Total effect 2 (Direct + Indirect through stage 2)	0.499 (0, 7.0 × 10^11^)	0.882 (0.438, 1.640)	0.906 (0.455, 1.783)	1.134 (0.443, 2.879)	1.835 (0.441, 7.707)
Total effect 3 (Direct + Indirect through stage 3)	0.519 (0, 6.82 × 10^10^)	0.885 (0.103, 6.069)	0.92 (0.079, 16.689)	1.036 (0.046, 14.348)	1.919 (0.186, 20.697)

**Table 4:** Bayesian analysis of DI on ovarian cancer survival mediated by cancer stage (3 levels). Each column represents a separate model with a different DI as exposure. Well-estimated hazard ratio shown in bold.

Gibbs Variable Selection for Covariates (modifiers). Covariates with Values Higher Than 0.5 Will Be Included in the Analysis			
Mediator: Stage	YOST	CDI	POV
Age	**1**	0.001	**0.972**
BMI	0.497	0.073	0.480
Self-reported SES	0.449	0.032	0.397
Smoking	0.485	**1**	0.491
Physical activity	**0.507**	0.029	**0.511**
Analysis result with selected modifiers. Each cell shows a posterior mean and its 95% credible interval			
Direct effect (Deprivation indices)	**0.736 (0.566, 0.954)**	1.036 (0.998, 1.075)	1.823 (0.777, 5.187)
Indirect effect Through stage 1	0.971 (0.073, 10.406)	0.971 (0.135, 7.717)	0.99 (0.242, 4.631)
Indirect effect Through stage 2	0.980 (0.292, 3.002)	0.989 (0.292, 3.369)	1.016 (0.413, 2.398)
Indirect effect Through stage 3	1.011 (0.101, 12.289)	1.01 (0.303, 3.504)	1.071 (0.124, 8.947)
Total effect 1 (Direct + Indirect through stage 1)	0.715 (0.050, 7.672)	1.006 (0.141, 7.987)	1.806 (0.345, 11.422)
Total effect 2 (Direct + Indirect through stage 2)	0.721 (0.211, 2.186)	1.025 (0.305, 3.474)	1.852 (0.598, 6.724)
Total effect 3 (Direct + Indirect through stage 3)	0.745 (0.073, 7.829)	1.047 (0.312, 3.616)	1.953 (0.154, 20.444)

**Table 5:** Bayesian analysis of DI on ovarian cancer survival mediated by debulking status (1 = Optimal debulking or CA125 after adjuvant <35; 2 = Suboptimal debulking or CA125 after adjuvant ≥35). Each column represents a separate model with a different DI as exposure. Well-estimated hazard ratio shown in bold.

Gibbs Variable Selection for Covariates (Modifiers). Covariates with Values Higher than 0.5 Will Be Included in the Analysis					
Mediator: Residual Disease	URB	MOB	SES	MICA	ADI
Age	**1**	0	**0.609**	0	**1**
BMI	0.499	0.007	0.109	0.1	**0.512**
Self-reported SES	0.392	0.002	0.020	0.004	0.369
Smoking	0.462	0	0.003	**1**	0.460
Physical activity	0.484	**1**	**1**	0	0.436
Analysis result with selected modifiers. Each cell shows a posterior mean and its 95% credible interval					
Direct effect (Deprivation indices)	**0.723 (0.592, 0.879)**	**0.883 (0.786, 0.999)**	**0.891 (0.812, 0.974)**	**1.205 (1.016, 1.459)**	**1.245 (1.040,1.491)**
Indirect effect Through level 1	1.039 (0.277, 4.232)	1.028 (0.096, 13.161)	0.980 (0.249, 3.756)	0.977 (0.148, 5.168)	1.016 (0.207, 6.387)
Indirect effect Through level 2	0.972 (0.213, 3.299)	1.001 (0.313, 3.072)	1.028 (0.332, 3.718)	1.009 (0.234, 4.694)	0.987 (0.399, 2.195)
Total effect 1 (Direct + Indirect through level 1)	0.751 (0.197, 3.14)	0.907 (0.086, 11.045)	0.873 (0.221, 3.366)	1.177 (0.166, 6.67)	1.265 (0.259, 7.898)
Total effect 2 (Direct + Indirect through level 2)	0.702 (0.150, 2.403)	0.884 (0.277, 2.708)	0.916 (0.299, 3.267)	1.216 (0.284, 5.561)	1.228 (0.494, 2.72)

**Table 6:** Bayesian analysis result of DI on ovarian cancer survival mediated by debulking status. (1 = Optimal debulking (or CA125 after adjuvant <35), 2 = Suboptimal debulking (or CA125 after adjuvant ≥35)). Each column represents a separate model with a different DI as exposure. Well-estimated hazard ratio shown in bold.

Gibbs Variable Selection for Covariates (Modifiers). Covariates with Values Higher Than 0.5 Will Be Included in the Analysis			
Mediator: Residual Disease	YOST	CDI	POV
Age	**1**	**1**	**1**
BMI	**0.50**	0.467	**0.510**
Self-reported SES	0.476	0.309	0.441
Smoking	0.480	0.454	0.485
Physical activity	0.495	0.409	0.494
Analysis result with selected modifiers. Each cell shows a posterior mean and its 95% credible interval			
Direct effect (Deprivation indices)	0.816 (0.664, 1.002)	**1.082 (1.026,1.147)**	1.751 (0.763, 4.518)
Indirect effect Through level 1	1.037 (0.149, 7.080)	0.993 (0.325, 3.271)	0.978 (0.116, 6.896)
Indirect effect Through level 2	0.978 (0.299, 3.111)	1.011 (0.162, 5.797)	0.996 (0.365, 2.820)
Total effect 1 (Direct + Indirect through level 1)	0.846 (0.123, 5.857)	1.075 (0.347, 3.545)	1.712 (0.195, 14.927)
Total effect 2 (Direct + Indirect through level 2)	0.798 (0.234, 2.565)	1.094 (0.176, 6.572)	1.744 (0.492, 6.091)

## Data Availability

The data used in this study can be made available to researchers once suitable IRB approval is obtained.

## Supplementary Materials

The following supporting information can be downloaded at: https://www.mdpi.com/article/10.3390/cancers15194848/s1. File S1: A summary of the approach is given here, with details of the approach and estimation method available in Supplementary Materials File S1.
