# Supplementary material for: The Association between Mediated Deprivation and Ovarian Cancer Survival among African American Women
Source: Cancers (Basel). 2023 Oct 4;15(19):4848. doi: 10.3390/cancers15194848 (PMC10571563; doi:10.3390/cancers15194848)
Supplement: Supplementary file 1 [file cancers-15-04848-s001.zip › cancers-2537278-supplementary.pdf]

Supplementary File S1

In this SM file the detail of the Bayesian joint mediation models is provided.

For a single mediator the following describes the model assumed: for the *i* th participant: the survival time is T_i, and the censoring indicator is δ_i. The mediator is defined to be M_i, the exposure is X_i, and the modifier set is $\{C_i\}$. In addition, we assume that a random effect at the participant level will be included in the models: U_i. For a survival outcome, we assume a conventional Weibull model, conditional on the mediator, exposure and observed and unobserved confounding:

$$T \mid X, M, \mathbf{C}, U \sim Weib(\exp(\beta_0 + \beta_x X + \beta_M M + \beta_C^T \mathbf{C} + \beta_U U), \nu)$$

This choice of model allows for a flexible form of hazard. In this formulation, the term $\beta_C^T \mathbf{C}$ is a linear predictor as a function of the modifier set.

For the mediation model, conditional on exposure and modifiers :

$$M \mid X, C, U \sim f(\alpha_0 + \alpha_x X + \alpha_C^T \mathbf{C} + \alpha_U U)$$

where $f(.)$ is a chosen mediator distribution. Note that the same random effect is shared and scaled in each of the outcome and mediator models.

Finally, the random effect model, which, in general, can depend on exposure and modifiers, is

$U \mid X, \mathbf{C} \sim g(.......X)$

where $g(.)$ is a suitable random effect distribution. This will be assumed to be continuous and zero mean Gaussian unless otherwise stated, and in our study will not be directly dependent on exposure or confounding. The above models are fitted jointly so that the random effect is shared but scaled. Note that the modifiers are assumed to have different linear predictors in the outcome and mediator models.

In this model structure, the direct and indirect effects of an exposure is defined as: Direct effect: $\approx \beta_x$, Indirect effect: $\approx \alpha_X \beta_M$ and Total effect: $\beta_X + \alpha_X \beta_M$.

Variable Selection

In the above model, the linear predictor for modifiers is assumed to be fixed. However, it is useful to consider an extension whereby we examine a range of putative models during our

estimation procedure. A Bayesian method which allows the sampling of all models within the computational method of MCMC is Gibbs variable selection. [21]

Within the design matrix $\mathbf{C}$ we can have multiple predictors and may wish to perform variable selection on these. Hence, we could replace $\beta_C^T\mathbf{C}$ and $\alpha_C^T\mathbf{C}$ with $\beta_C^{*T}\mathbf{C}$ where $\beta_C^* = \beta_c.\lambda_c^S$ and $\alpha_C^{*T}\mathbf{C}$ where $\alpha_C^* = \alpha_c.\lambda_c^M$ respectively. The λparameters are inclusion or entry parameters and allow the predictors to be swapped in and out of the model during estimation. It is assumed that the ‘entry’ parameters have Bernoulli prior distributions: $\lambda_{cj}^S \sim Bern(p_{cj}^S)$, $\lambda_{cj}^M \sim Bern(p_{cj}^M)$. This allows the inclusion parameters to range between [0,1]. When the estimation process is complete, the inclusion parameters are averaged and any predictor where $ave(\lambda) > 0.5$ is selected for a final model. [22] The final model is then fitted with only those predictors reaching this threshold.

Computation and Prior Distributions

To fit the above model, and all the model variants we discuss here, we have implemented the models using a MCMC procedure in the R package Nimble.[23] In general, we assume regression parameters to have conventional zero mean Gaussian prior distributions with precisions which have *Ga(a,b)* prior distributions. We assume weaky informative gamma priors with a = 2.0 and b = 0.5.

Nimble chooses the most appropriate sampling algorithms to use to generate samples from the posterior distribution.
